# Empirical and conceptual investigation of de-implementation of low-value care from professional and health care system perspectives: a study protocol

**DOI:** 10.1186/s13012-018-0760-7

**Published:** 2018-05-15

**Authors:** Henna Hasson, Per Nilsen, Hanna Augustsson, Ulrica von Thiele Schwarz

**Affiliations:** 1 Procome research group, Medical Management Centre, Department of Learning, Informatics, Management and Ethics, Karolinska Institutet, SE 171 77 Stockholm, Sweden; 2 Unit for implementation and evaluation, Center for Epidemiology and Community Medicine (CES), Stockholm County Council, SE 171 29 Stockholm, Sweden; 3 Department of Medical and Health Sciences, Division of Community Medicine, Linköping University, Linköping, Sweden; 4 School of Health, Care and Social Welfare, Mälardalen University, Box 883, 721 23 Västerås, Sweden

**Keywords:** Disinvestment, Unlearning, Habits, Ineffective, Abandon, De-adoption, Decrease use

## Abstract

**Background:**

A considerable proportion of interventions provided to patients lacks evidence of their effectiveness. This implies that patients may receive ineffective, unnecessary, or even harmful care. Thus, in addition to implementing evidence-based practices, there is also a need to abandon interventions that are not based on best evidence, i.e., low-value care. However, research on de-implementation is limited, and there is a lack of knowledge about how effective de-implementation processes should be carried out. The aim of this project is to explore the phenomenon of the de-implementation of low-value health care practices from the perspective of professionals and the health care system.

**Methods:**

Theories of habits and developmental learning in combination with theories of organizational alignment will be used. The project’s work will be conducted in five steps. Step 1 is a scoping review of the literature, and Step 2 has an explorative design involving interviews with health care stakeholders. Step 3 has a prospective design in which workplaces and professionals are shadowed during an ongoing de-implementation. In Step 4, a conceptual framework for de-implementation will be developed based on the previous steps. In Step 5, strategies for de-implementation are identified using a co-design approach.

**Discussion:**

This project contributes new knowledge to implementation science consisting of empirical data, a conceptual framework, and strategy suggestions on de-implementation of low-value care. The professionals’ perspectives will be highlighted, including insights into how they make decisions, handle de-implementation in daily practice, and what consequences it has on their work. Furthermore, the health care system perspective will be considered and new knowledge on how de-implementation can be understood across health care system levels will be obtained. The theories of habits and developmental learning can also offer insights into how context triggers and reinforces certain behaviors and how factors at the individual and the organizational levels interact. The project employs a solution-oriented perspective by developing a framework for de-implementation of low-value practices and suggesting practical strategies to improve de-implementation processes at all levels of the health care system. The framework and the strategies can thereafter be evaluated for their validity and impact in future studies.

## Background

The importance of basing care practices on evidence and empirical research findings rather than on experience or beliefs is widely accepted in health care [1]. However, putting evidence from research into practice has been shown to be challenging, as indicated by the renowned research–practice gap [2]. This gap tends to be approached from the perspective of research (i.e., that there are efficient interventions that need to be implemented). However, another aspect of this gap is that some health care practices (e.g., interventions, programs, and services) lack evidence of effectiveness and may even be harmful. Such low-value care practices, i.e., practices which lack current, best evidence, should therefore be abandoned, which is often referred to as de-implementation [3].

Use of low-value care practices is common. Estimates show that 12–15% of patients receive at least one low-value practice a year [4], and 72% of physicians in a study conducted in the USA stated they prescribe unnecessary tests or procedures at least once a week [5]. The most common type of low-value care is likely an inappropriate use of an effective practice for patients for whom the practice’s benefit has not been demonstrated [6]. The yearly cost of low-value care was an estimated $8.5 billion for the USA Medicare population, which constitutes almost 3% of the total Medicare annual spending on services [7]. Thus, the considerable prevalence and unnecessary costs of the use of low-value care have made this a major problem for health care systems.

While implementation of evidence-based practices has received substantial attention, the challenges of de-implementation of low-value care have so far attracted limited interest from researchers, professionals, and decision- and policy-makers [3, 8]. Lists of non-recommended practices such as Choosing Wisely [6] have become common, yet they have little chance of leading to improvements without sufficient knowledge about efficient de-implementation, i.e., translating the “what” should be de-implemented into the “how” this actually should be carried out [6, 9–12].

The challenges of de-implementation decisions and processes can be considered from both the perspectives of health care *professionals* and the *health care system*. For health care professionals, de-implementation involves ceasing to do something. This might require unlearning and breaking ingrained habits, which tends to be difficult [13–15]. It can be a threatening process to challenge well-established, entrenched thoughts and action patterns [16], particularly for those invested in that practice. Various practices can be deeply embedded in a person’s position, status, values, and identity. Abandoning a practice can therefore be emotional, touching upon issues such as what is considered valuable knowledge, ethics, and professional roles and expectations. Thus, de-implementation may act as a cognitive stressor by evoking contradictory demands. It may also act as an emotional stressor, causing ethical distress in instances, for example, when professionals know that practice is not recommended any longer but their role or status is associated with that specific practice. Questions concerning how health care professionals deal with de-implementation, what responses they have to de-implementation, and what influences this process have not yet been systematically investigated. Hence, there is a need for research about de-implementation from health care professionals’ perspectives.

Stopping certain practices often requires decisions at many levels, from the group, department, and organizational levels to regional and national levels. Thus, de-implementation of low-value practices is an issue at all levels of the health care system [17–19]. Prevailing norms, values, work processes, and financial and professional interests play crucial roles in de-implementation decisions and processes [12, 20]. This implies that the decisions of individual professionals often depend on the culture of their professional groups and/or the decisions made at other levels of the system. Thus, research on de-implementation requires a system-level perspective. However, research on how de-implementation decisions and processes are managed in health care is limited.

Factors that influence implementation are well-known, having been identified in many empirical studies and synthesized in numerous so-called determinant frameworks [21], e.g., Consolidated Framework for Implementation Research [22], Promoting Action on Research Implementation in Health Services [23], and Theoretical Domains Framework [24]. These frameworks categorize a number of interdependent determinant domains encompassing different levels, from the individual professional, team, and department to the organizational and societal levels. However, the extent to which the same or similar factors or domains also influence decisions and processes concerning de-implementation of low-value practices has not been investigated.

There is some empirical research that points to the importance of individual factors for de-implementation decisions and processes. For instance, it has been shown that physicians’ use of low-value care is strongly influenced by patient preferences and wishes. Over half of US physicians reported that they would order tests if they were requested by a patient even if they knew these tests were unnecessary [5]. Fear of malpractice or missing an important diagnosis was the main reason, implying that the tests, although low-value in specific patient cases, offered a sense of security for the physicians. Furthermore, physicians’ perceptions of the importance of de-implementation are influenced by how convincing the lack of evidence for the low-value practice is and whether there are alternative practices available [25]. Physicians have also shown uncertainty in their knowledge to interpret research findings, which causes difficulties in their ability to make decisions about abandoning low-value care [26].

Concerning the organizational and other system levels, empirical research thus far suggests that de-implementation decisions and processes are affected by factors such as political interests, invested interests, and community expectations together with media engagement [8]. There is also a risk that cost considerations are prioritized over effectiveness [25]. Furthermore, steering mechanisms, including financial incentives, may influence the practices being used. For instance, fee-for-service payment models may incentivize the use of low-value care practices [27]. Furthermore, a lack of decision-support systems, poor quality of data, and poor communication and leadership potentially influence de-implementation of low-value practices [8, 26, 28]. Shepperd et al. [26] found that administrative health care managers in the UK in general lacked understanding and knowledge about how to efficiently de-implement low-value care. They also emphasized the importance of social processes with sense-making and goal clarification to succeed with de-implementation processes. In practice, decisions to stop use of low-value care were often processes that lacked clear structures, with opportunities to monitoring the success being few [26]. In sum, existing empirical research suggests that the individual level and many system levels are intertwined in de-implementation decisions and processes.

The aim of this project is to investigate de-implementation decisions and processes concerning low-value health care practices from the perspective of professionals and health care systems.

Specific research questions:

1. How is de-implementation described in the literature concerning (a) determinants influencing de-implementation decisions and processes, (b) interventions that have been used for de-implementation, and (c) existing frameworks/models for de-implementation?
2. How do health care actors (e.g., professionals, managers and administrative management) reason about the de-implementation of low-value care practices, and how can variation in de-implementation practices be understood?
3. How do health care professionals approach de-implementation processes in practice, and what are the cognitive, emotional, and behavioral responses to these de-implementation processes?
4. How can factors that influence de-implementation decisions and processes be categorized and synthesized into a conceptual framework?
5. How can de-implementation be facilitated at different health care system levels (national, regional, organizational, and individual)?

This project will apply theories of relevance for understanding and explaining de-implementation at different system levels, from the individual to the societal. Theories concerning habits and developmental learning in combination with theories of organizational alignment will be used. Health care professionals perform hundreds of tasks each workday. Many of these are executed habitually, that is, without deliberation or conscious awareness [29]. Habits are highly necessary for the individuals and the system but become problematic when the habitual behaviors need to be modified, as in the case of no longer using low-value practices [30].

According to developmental learning theory, the first step in ceasing habitual behavior is making its practitioners conscious of it [31]. This can be done, for instance, through education or information. Yet, mere knowledge is rarely sufficient for changing behaviors [32]. From theories on developmental learning and habits, this can be understood in at least two ways: (1) It requires more time, effort, and motivation to change habitual behavior than to continue doing what normally is done and (2) the habitual behaviors are embedded in a context, which has many cues that elicit the behaviors in question, whereas there are few or no cues for an alternative, new behavior [30, 31]. The latter links the theories of individuals’ habits to organizational theories, indicating why habits are context-dependent [33, 34].

The theory of alignment describes how organizational behaviors and practices need to be aligned with the overall goals, systems, and functions that are in place [34]. This implies that individuals’ opportunities, motivations, and abilities for abandoning their work practice habits are dependent on the expectations, norms, and values in the organizational context. The organizational context, i.e., what is appreciated, possible, encouraged, supported, applauded, and rewarded at a workplace, can often be expected to have greater impact on individuals’ behaviors than more abstract factors such as scientific knowledge, clinical guidelines, or other recommendations of care practices [32].

The process of habits goes the other way around, as well: The habits of the individual become the routines of the collective, emphasizing the interaction between the individual and the context in which he or she works. The routines become norms, representing the unwritten rules for a profession (e.g., physicians, nurses) or other social groups (e.g., work units) when these are socially approved and shared. Norms in turn provide a sense of common direction [34–36]. The powerful impact the nearest organizational culture has on health care professionals behaviors when it comes to implementation of new knowledge has been confirmed in numerous studies from implementation science [22, 37, 38]. Thus, the surrounding social system provides strong cues for behaviors.

## Methods

This project has multiple research designs and consists of five main steps, which correspond to the five research questions. Step 1 is a scoping review of the literature. Step 2 has an explorative design involving interviews with health care stakeholders. Step 3 has a prospective design in which workplaces and professionals are shadowed during an ongoing de-implementation. In Step 4, a conceptual framework for de-implementation will be developed based on the previous steps. In the 5th step, strategies for de-implementation are identified using a co-created design approach.

The study will be conducted in Stockholm County Council in Sweden. Sweden has 20 county councils/regions that are responsible for provision of health care in their regions. Stockholm County Council is the largest one in the country. It provides all types of care and health care locations, including acute care hospitals, psychiatric care, primary health care centers, and rehabilitation. Stockholm County Council health care organization has approximately a total of 36,000 employees. Health care in Sweden is largely publicly funded. All residents are insured by the state and have equal access to health care. Out-of-pocket fees are low and regulated by law.

### Step 1—scoping the literature on de-implementation

This step involves a scoping literature review to understand how de-implementation is described in the scientific literature. The scoping review will follow the steps outlined by Arksey and O’Malley [39]. Three research questions will be addressed: (a) which determinants for de-implementation decisions and processes in healthcare have been described, (b) which interventions have been used for de-implementation in healthcare, and (c) which frameworks/models for de-implementation have been described? Relevant studies will be identified through searches in electronic databases (i.e., Embase, MEDLINE, and Web of Science) and through searches of reference lists and of key journals, such as the Implementation Science. Two researchers will review abstracts and full articles to determine inclusion or exclusion. Studies in English that address one or more of the research questions will be included in the review. Studies focusing solely on prevalence of overuse will not be included. The inclusion criteria for studies included will be developed during the course of the process (rather than strictly pre-defined) [39]. In the next step, key information will be extracted from the articles and entered into a data extraction form. Extracted information will be both general, for example, study setting and population, and specific, addressing the research questions, e.g., determinants found to influence de-implementation or interventions used to facilitate de-implementation. Finally, the data will be summarized, collated, and reported. An overview of the extent, nature, and distribution of the studies in the review will be presented. The information from the studies will be organized thematically and summarized in order to respond to the research questions. The themes will be inductively developed based on the empirical data and determined during the analytical process.

### Step 2—describing health care actors’ reasoning concerning de-implementation

This step involves a qualitative data collection which will be carried out in accordance with the COREQ checklist [40]. Semi-structured interviews will be conducted to understand how health care actors (e.g., professionals, managers, and administrative management) reason through de-implementation and how variation in de-implementation practices can be understood. We will start with identifying two to three low-value care practices (i.e., methods that should be de-implemented) through key stakeholders at the Stockholm County Council. This includes the local Health Technology Assessment organization, senior managers, frontline professionals, and individuals with a formal responsibility for medical, nursing, or other areas of care (e.g., chief medical officers). When a specific low-value care practice has been identified, the managers of all work places in the Stockholm County Council where this practice is or has been used will be approached. The managers are asked to forward an invitation to the interviews for individuals who have knowledge and experience relating to the specific practice. Those individuals responding to the invitation will be approached by the research team with a clarification of the interview aim and practical details. Thereafter, we will also use snowballing to recruit additional stakeholders in the same and other respondent groups (different professionals, managers, administrative management). Additionally, during the interviews, we will ask interviewees to identify additional low-value care practices that have been or should be de-implemented. The participants will vary in terms of professions, their positions in the health care system, the low-value care practices they have experience with, and the health care settings. This variation is sought to achieve a richer variation of de-implementation. We hypothesize that decisions and processes differ between the low-value care practices. Thus, the participant selection is purposive sampling and the recruitment will continue until multi-level saturation is achieved; that is, we strive for sufficient information across professional groups, work units, and de-implementation cases. We estimate that this will require around 30–50 interviews.

An interview guide will be developed based on the guiding theories and the literature review. As de-implementation might be perceived as a delicate subject, building confidence between the researchers and the respondents will be of importance to ensure that the interviews reflect reality. We attempt to establish this by using researchers with extensive experience in interviewing as well as in evidence-based practice in health care. Two members of the research team will be responsible for the data collection and analysis. The remaining research group will act as informed outsiders. They will participate in iterative debriefing sessions to support the analysis and interpretation of the findings. Thus, data collection, transcription of recordings, and analysis will be done iteratively. A hypo-deductive approach following the step outline by Fereday et al. [41] will be used to analyze the data. We will start with the hypothesis from the theories of habits, developmental learning, and alignment and test these in several steps with the data. The interviews will be audiotaped and transcribed verbatim. The nVivo software will be used for the data analysis.

### Step 3—following professionals’ approaches to de-implementation

This step is a prospective, longitudinal case study focusing on how health care professionals approach a de-implementation. A case will be identified primarily from the Swedish National Board of Health and Welfare’s “not to do-list” [42] and other guiding documents (e.g., clinical practice guidelines and regional care programs) in collaboration with health care stakeholders. There will be three national clinical guidelines including “not to do-lists” from the Swedish National Board of Health and Welfare during 2018. The final selection of a case from one of these guidelines will reflect a low-value practice that is common and will involve several professional groups and in which there has been a clear formal decision to de-implement that practice.

After deciding which low-value practice that will be studied, work units within the Stockholm County Council where this practice is used will be approached. An invitation to participate will be sent to the managers of those units. The de-implementation process will be followed over a period of 12 months in all, or a selection of, work units. We estimate that two to four work units will be needed to receive sufficient variation in a de-implementation process. The balance between the number of work units and the number of individuals within work units will be determined based on information about variation derived from steps 1 and 2: If most of the variation in de-implementation determinants exists at the individual level, we will recruit more individuals and fewer work places, and vice versa. The data will be iteratively collected and analyzed, which will allow us to observe potential data saturation across individuals and units. We will start with two work units and at least eight individuals at each work place, reiteratively collecting and analyzing data and expanding the data collection until multi-level saturation is reached. Health care professionals from all professional groups involved in the use of the specific work practice will be the main informants.

Multiple data sources will be used to capture the de-implementation process. Structured observations will be complemented with interviews and collection of documents concerning an ongoing de-implementation process [43]. Observations will be focused on staff meetings and other work situations where de-implementation may be discussed, and paper trails such as minutes of meetings and other documentation will be gathered. No data where patients can be identified will be collected. The observations will be conducted in accordance to a structured process, using a pilot-tested protocol [44, 45]. The protocol will indicate time and the activity’s pre-set agenda, content (e.g., what is verbalized by whom in speech or writing), and the process (e.g., types of activities and procedures) and other potential categories of interest based on the guiding theories and the literature review. The observer makes field notes in the protocol during or after each observation. The complementary interviews will be based on the observations and enable the researchers to ask follow-up questions based on previous observations. An interview guide will be developed but allow flexibility based on the observational findings. One member of the research team will carry out all data collection at this step and is responsible for the analysis. As with the previous step, the remaining research group will support the analysis and interpretation by acting as informed outsiders in iterative debriefing sessions. The multiple source data will be triangulated. The interviews will be audiotaped and transcribed verbatim. The nVivo software will be used for the data analysis.

### Step 4—developing a conceptual framework

Addressing research question 4, this step entails development of a conceptual framework to describe and categorize various influences on de-implementation, (determinant domains), including individual- and system-level determinants. The framework will be based on the literature review and findings from the empirical data from the previous steps. A comparison will also be made with one of the existing determinant frameworks for implementation. We have explored numerous potential frameworks, many of which include the same or similar determinants [21]. We plan to use the Consolidated Framework for Implementation Research (CFIR) [22–24], since it is one of the most comprehensive frameworks. It is also sufficiently broad to allow for an explorative, inductive approach to collecting data. This approach is important since there are few studies which have investigated and attempted to categorize determinants of de-implementation. Furthermore, CFIR is the most widely used determinant framework by implementation researchers, according to an international survey of 223 implementation researchers [46]. A preliminary version of the conceptual framework for de-implementation will be reiteratively tested with health care stakeholders as well as national and international collaborating researchers to obtain optimal scientific rigor and practical usefulness.

### Step 5—identifying strategies for de-implementation

In this step, strategies will be developed and feasibility-tested to address how de-implementation can be facilitated at various health care system levels to ensure that an effective process is achieved. The framework developed in step 4 will be the foundation for the strategies and international and national experts on de-implementation, and stakeholders involved in the previous steps will be invited to participate in workshops to develop the strategies. A structured process, the co-created program logic (COP), will be used to synthesize knowledge about strategies for de-implementation from the previous steps and across the different participants [47]. This method is used to utilize knowledge and experience from multiple sources and create a shared understanding among the participants. COP is flexible in terms of number of participants; the final number will be decided based on the number of relevant participants that can be identified. Following the COP process, the stakeholders will be asked to rate the feasibility and importance of each strategy. Descriptive statistics will be used to analyze the data from the feasibility questionnaires.

## Discussion

This project has the ambition to make substantial contributions to implementation research and practice with regard to de-implementation of low-value care, thus addressing the well-known gap between research and practice. First, it contributes via empirical data on how de-implementation of low-value practices is handled by professionals. This offers insights into how professionals make decisions and reason through de-implementation in daily practice and the consequences this has on their work. Importantly, de-implementation will be investigated from the perspective of several professional groups (rather than solely focusing on physicians as has been common in previous studies) and line managers (in addition to administrative management, which has been the focus of prior studies). Thus, the current project has the potential to contribute to a deeper understanding of de-implementation of low-value practices in the health care setting.

Second, the health care system perspective on de-implementation of low-value care will be highlighted. Stopping certain work practices might require decisions and actions at multiple levels, from the team or unit to the organizational, regional, and national levels. The project will contribute to knowledge of how de-implementation can be understood across health care system levels, which is important in facilitating effective processes across the whole system. The importance of contextual perspectives in implementing new knowledge has increasingly been emphasized in implementation research [22, 37, 48].

A third contribution concerns the project’s use of theories of habits, developmental learning, and organizational alignment. The relevance of these theories has been suggested [29] but few empirical studies that apply them have been conducted. Theories of habits and developmental learning can offer insights into how context triggers and reinforces certain behaviors (but not others) and how factors at the individual and the organizational levels interact. The theories will be used to analyze how different factors influence health care professionals’ continued use of low-value practices that should be abandoned due to their low value and to explore what cues and opportunities are needed to stimulate abandonment of such practices. The theoretical approach of the project facilitates insights into how and why certain factors have impact rather than merely producing lists of factors having impact. Fourth, the project aims to develop a conceptual framework for de-implementation of low-value practices. This can be used as a tool in future studies that aim to systematically approach de-implementation. The framework will illustrate all levels of the health care system in relation to abandonment of low-value care practices. Fifth, the project has the ambition to develop theory-based strategies to improve de-implementation processes at all levels of the health care system. Because de-implementation is an under-researched area, there are few solutions available for researchers and practitioners. Therefore, this project contributes potential solutions that the research community thereafter can evaluate for their impact in future studies.

This project is explorative in character due to limited knowledge about the phenomenon of interest, i.e., de-implementation in health care. The project begins with a study (Step 1) aimed at achieving improved understanding of the concept of de-implementation and the terms used to denote this. The other steps explore the determinants of de-implementation, and the final step is aimed at identifying potential strategies that can be used to improve the process. However, the project does not involve any intervention evaluations, which will be an important next step after more knowledge has been generated.

A scoping review methodology has been chosen since the research field is still immature. This kind of review is valuable in exploring what kind of research has been done in an area, and unlike in systematic reviews, the question is often wider, and studies with all types of designs are included [39]. The literature review is expected to offer a broad understanding of the key studies and concepts underpinning the field. An important strength of the project is the combination of different methodological approaches (literature review, empirical data, conceptual work, and strategy development). We will be able to gain insights into de-implementation by using both empirical and conceptual perspectives. Furthermore, several levels of the health care system are addressed, which is important for understanding the interplay between individuals (e.g., habits), organizations (e.g., norms and climates), and health care systems (e.g., rules and regulations).
